# The effect of a lidocaine/prilocaine topical anesthetic on pain and discomfort associated with orthodontic elastomeric separator placement

**DOI:** 10.1186/s40510-016-0156-7

**Published:** 2017-01-09

**Authors:** M. Abu Al-Melh, L. Andersson

**Affiliations:** 1 Department of Developmental and Preventive Sciences, Faculty of Dentistry, Kuwait University, Kuwait city, Kuwait; 2 Department of Surgical Sciences, Faculty of Dentistry, Kuwait University, Kuwait city, Kuwait

**Keywords:** Pain, Anesthetics, Separators, Orthodontics, Topical

## Abstract

**Background:**

The initial placement of orthodontic elastomeric separators can be uncomfortable and painful. Therefore, it is important to relieve this disturbing sensation to create a discomfort or pain-free orthodontic visit. The purpose of this study was to investigate the effect of a lidocaine/prilocaine topical anesthetic on pain and discomfort associated with the placement of orthodontic elastomeric separators.

**Methods:**

Fifty subjects aging between 20–35 years were included in this study. In the maxillary arch, a lidocaine/prilocaine topical anesthetic was placed around the ginigval margins of the premolar and molar on side. On the other side, a placebo agent was placed around the ginigval margins of the premolar and molar. After two minutes, an elastomeric separator was placed between the premolar and molar on both sides. The subjects were then asked to report their findings on a Verbal Scale and a Visual Analogue Scale every second minute for a period of 10 min. The subjects were also given a questionnaire to evaluate the overall impression on the topical anesthetic use.

**Results:**

The overall mean discomfort/pain score was found to be significantly lower (*p* < 0.001) with the topical anesthetic than with the placebo. Repeated measures ANOVA with a Greenhouse-Geisser correction determined that mean pain scores were statistically significantly low with the 10-min time duration (*F*_(1.54,42.2)_ = 40.7, *p* = 0.001), with an estimated grand mean (8.37, 95% CI 6.75–9.98). The questionnaire responses revealed that 87% of the subjects reported an overall satisfaction and agreement with the topical anesthetic than with the placebo or no difference (13%) after the initial separator placement.

**Conclusions:**

The discomfort and pain resulting from the initial placement of orthodontic elastomeric separators can be significantly reduced with the lidocaine/prilocaine topical anesthetic.

## Background

Orthodontic discomfort and pain can be disadvantageous to the patient’s compliance and response to treatment. This unpleasant experience may occasionally cause loss of interest, poor compliance, compromised treatment results, and even eventual termination of treatment [1, 2]. The various painful and distressing orthodontic procedures include separator placement, archwire insertion and activation, application of orthopedic forces, use of elastics, and debonding procedures [3]. The accompanying uncomfortable sensations experienced by patients during orthodontic treatment are often described as feelings of pressure, tightness, soreness of the teeth, and pain [4].

It was demonstrated in some studies that amongst the discomfort and pain causing orthodontic procedures is the placement of separators, which can be elastomeric, brass wire, spring type, steel, and latex separators [4–6]. These studies have reportedly shown that discomfort was clearly associated with separator placement. The sensation of discomfort often starts within 4 h of separator placement, gradually increasing over the next 24 h, and then tends to decrease within 7 days [4, 5]. The orthodontic patients’ response to the initial placement of separators seems to be overlooked, and there is no current information in the literature that analyzed the patients’ initial response to such a procedure.

There are several methods for managing orthodontic pain and discomfort that were covered in the literature. The most common method used to manage orthodontic pain and discomfort was the use of systemic analgesics [5, 7–10]. Chewing on something hard such as a chewing gum or a plastic wafer, during the first few hours of appliance activation, has been recommended for reducing the orthodontic pain [11–14]. Other methods such as low-level laser therapy (LLLT), transcutaneous electrical nerve stimulation (TENS), and vibratory stimulation were also advocated for managing orthodontic pain [13, 15–17].

Topical anesthetics have been used in different dental procedures for reducing or eliminating pain. It was demonstrated that pain from needle stick injections in the maxillary vestibular and palatal mucosae could potently be reduced or eliminated by using a combination of 2.5% lidocaine/2.5% prilocaine topical anesthetics in a creamy eutectic mixture, known as EMLA® (EMLA, AstraZeneca UK Limited, Bedfordshire), or a thermosetting gel, known as Oraqix® (ORAQIX, DENTSPLY International, PA, USA) [18–20]. Other studies reported different useful applications of lidocaine, adrenaline, and tetracaine (LAT) gel and EMLA® cream, such as suturing of the facial and soft tissue lacerations and minor biopsies [17, 21–23]. One study concluded that lidocaine and prilocaine topical anesthetics could be used in oral mucosal lacerations prior to suturing without the risk of adverse tissue reaction [24].

The effect of the lidocaine/prilocaine topical anesthetic Oraqix® on pain reduction from orthodontic procedures has been studied previously. The findings of one study suggested the potential usefulness of Oraqix® in performing orthodontic procedures such as band placement and cementation, archwire ligation, and band/bracket removal [25]. The advantage of the topical anesthetic gel Oraqix® was its delivery method, which simply introduced the gel into the gingival crevice. The suggested indication for use was correlated with the reduction of pain during scaling in gingival pockets. The gel hardened with intraoral temperature and hence was easily contained within the gingival crevice. Also, the application procedure was reportedly simple and completely painless [26]. Since Oraqix® has not yet been used for discomfort and pain relief from the initial elastomeric separator placement, extending the use of Oraqix® to relieve patients from the associated sensation of discomfort would be an interesting achievement.

This study was aimed at comparing the topical anesthetic effect of a 2.5% lidocaine/2.5% prilocaine gel (Oraqix®) with a Vaseline® placebo on the reduction of discomfort and pain from the initial placement of orthodontic elastomeric separators.

## Methods

Fifty subjects, between 20 and 35 years of age, were included in this study, 47 females and 3 males. The subjects were undergraduate fifth- and sixth-year dental students, staff members, and dental assistants from the Faculty of Dentistry, Kuwait University. A written consent was obtained from all subjects participating in this study. The study’s experimental design and protocol were approved by the Ethical Committee of the Health Sciences Center, Kuwait University.

The inclusion criteria of this study involved the presence of healthy gingival tissues, complete intact posterior occlusion, intact maxillary dentition with the exception of the third molars, and tight contacts between the posterior teeth which was checked with a piece of floss. The exclusion criteria comprised of the existence of inflamed gingival tissues and periodontal disease, missing posterior teeth, spacing between the posterior dentition, retained deciduous posterior teeth, and interproximal carious lesions and/or restorations between the first molar and the second premolar. Subjects with systemic diseases and/or are taking systemic analgesics were excluded from the study.

Only the subjects were blinded during the study by wearing sunglasses with gauze taped to the inner side of the shades. The gingival tissues of the first molars and the second premolars from both sides of the maxillary arch were first dried using gauze and the air-water syringe. A suction device and cotton rolls were used to achieve a dry field prior to the application of agents and throughout the procedure. The subjects had their mouths open during the entire experimental procedure. This was done to prevent accidental distribution of the topical anesthetic, Oraqix®, to the placebo, Vaseline®, side.

**Fig. 1:**
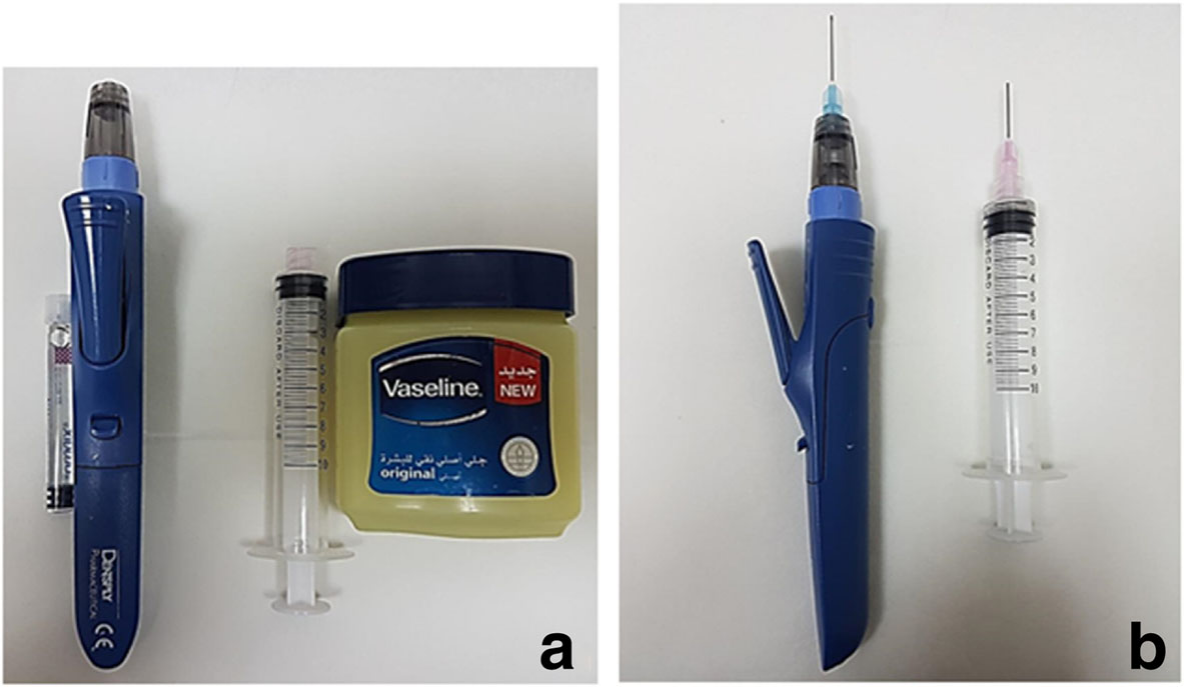
**a** Side-by-side view of the materials used: on the *right side*, the Oraqix® vial and its dispensing syringe, and on the *left side*, the Vaseline® and the syringe used for its application. **b** Side-by-side view of the syringes loaded with the materials and the blunt application needles installed

**Fig. 2:**
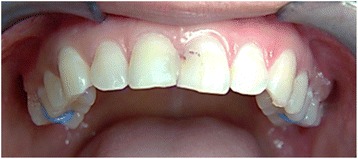
Intraoral photograph. An upper frontal view of the maxillary arch showing the materials placed around the gingival margins and into the crevices of the maxillary first molar and second premolar on both sides. On the *upper right side*, the topical anesthetic (TA) Oraqix® was placed. Oraqix® is a transparent and colorless material which is difficult to view in the photograph. On the *left side*, the placebo (Vaseline®) was placed

A split-mouth design was applied. On one side, using an Oraqix® dispenser and a blunt dispensing needle, the Oraqix® gel was injected around the gingival margins and into the crevices of the first molar and the second premolar (Figs. 1 and 2). The procedure of dispensing the topical anesthetic gel was non-invasive and entirely painless as the gel was applied directly to the soft tissue of the periodontal pocket, and it was allowed to saturate into the gingival crevice.

A few drops of Oraqix® were applied on the orthodontic elastomeric separator (Ormco Separators, Ormco Corporation, CA) prior to its placement between the teeth to ensure that the topical anesthetic agent reached the interproximal tissues adequately without the need to use a dispensing needle, which may cause discomfort or pain. Moreover, Oraqix® is a liquid gel when first applied, which is difficult to contain within the gingival tissues, unlike Vaseline® (Vaseline®, 100% pure petroleum jelly, Unilever, USA), which is more viscous and easily contained on and within the gingival tissues. In addition, placing a few drops of Oraqix® on the separator before its placement between the teeth did not facilitate its insertion as the contact points between the teeth which were tight to begin with.

On the contralateral side, using an irrigation syringe with a blunt applicator tip, a small size amount of placebo Vaseline® was placed around the gingival margins of the first molar and the second premolar (Figs. 1 and 2). The sides, where the materials were applied, were randomly alternated in such a way that if the first subject received Oraqix® on the right side, then the next subject had it on the left side and so on. At the end of the study, half of the subjects received Oraqix® on the right side and the other half had it on the left side.

**Fig. 3:**
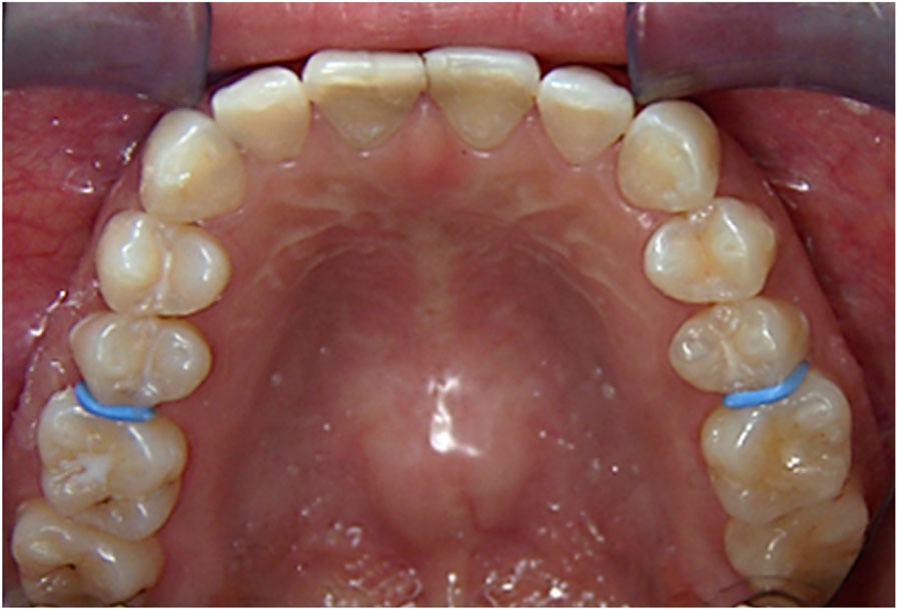
Intraoral photograph. An upper occlusal view showing the orthodontic elastomeric separators placed between the maxillary second premolars and first molars

After 2 min from the application of the agents on both sides, the orthodontic elastomeric separator was stretched, using two pieces of floss, and placed between the first molar and the second premolar on both sides (Fig. 3). The subjects were requested to immediately report the degree of pain on both a visual analogue scale (VAS) and a verbal scale. The overall pain was measured by the subjects by means of a 100-mm horizontal non-graded VAS, with the left endpoint marked as “no discomfort/pain,” and the right endpoint marked “worst possible discomfort/pain,” as the primary efficacy parameter. A verbal rating scale, which permitted the subject to make a direct comparative assessment on asking which side was least painful, was used as a secondary efficacy parameter. The subjects had three choices to select from that included “right side,” “left side,” or “no difference.” The subjects’ responses were recorded every second minute for a total period of 10 min. At the end of the study, the separators were removed and the subjects were asked to rinse their mouths with water to wash off the materials applied.

A questionnaire was given to the subjects and was returned the following day. The survey contained comparative questions about the overall satisfaction, taste, numbing effect, presence of numbness after 30 min, personal preference, recommendation for routine use in orthodontic clinics, recommendation for application on adults and children, and the experience of any adverse reactions the following day.

### Statistical analysis

The data management, analysis, and graphical presentation were carried out using the computer software, “Statistical Package for Social Sciences, SPSS version 22.0” (IBM Corp., Armonk, NY, USA). The descriptive statistics for all the subjects reporting least pain sensation when comparing both sides have been presented as numbers and percentages, and the continuous variable, percent pain sensation as mean ± standard deviation (SD). The general linear model (GLM) for repeated measures ANOVA was applied to see within-subject effect at five different durations of time, from 2 min until 10 min, as well as the between-subject factor, placebo (Vaseline®) and topical anesthetic (Oraqix®). Greenhouse-Geisser correction was applied as the Mauchly’s test of sphericity was not met. The estimated marginal means are also reported with 95% confidence interval (CI). The two-tailed probability value *p* < 0.05 was considered statistically significant.

## Results

### Comparison of visual analogue scores (VAS)—Table 1 and Figs. 4 and 5

**Table 1:** Mean percent pain scores on visual analogue scale (VAS) with estimates ANOVA with repeated measures (RM)

Duration (minutes)	Number	Mean ± SD (TA)	Mean ± SD (Placebo)	Estimated grand mean (95% CI)	*p* value (ANOVA RM)
2	50	11.53 **±** 11.27	20.91**±**20.74	16.22 (12.91–19.53)	
4	50	4.52 **±** 7.40	14.99**±**13.23	9.76 (7.63–11.88)	*F* = 40.7
6	50	2.65 **±** 6.10	11.79**±**11.13	7.22 (5.44–9.00)	(*p* = 0.001)
8	50	0.80 **±** 2.79	8.90 **±** 9.37	4.85 (3.48–6.22)	
10	50	0.30 **±** 0.95	7.27**±**10.12	3.78 (2.36–5.12)

**Fig. 4:**
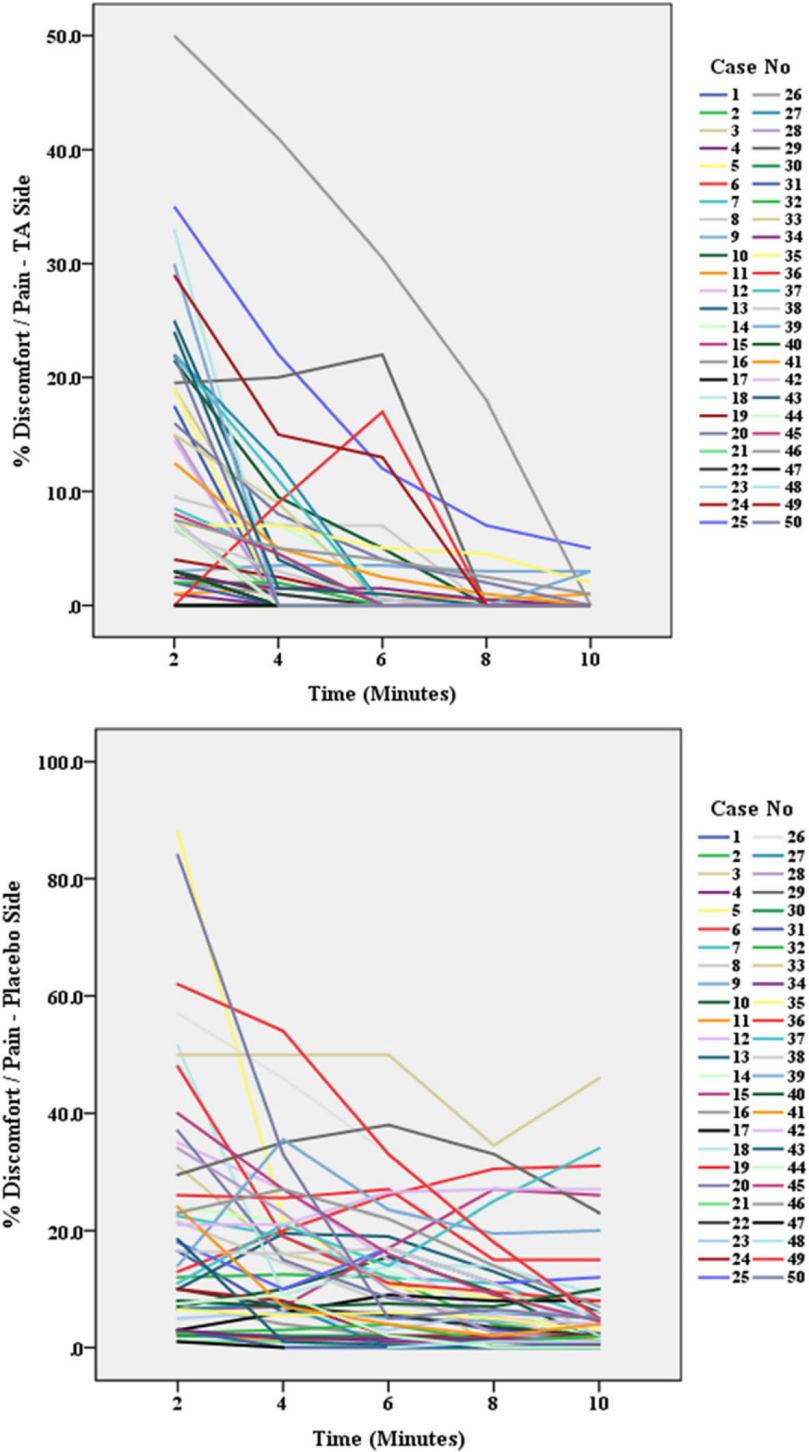
Visual analogue scale (VAS). VAS scores (%) at different times (minutes) after the application of topical anesthetic (TA Oraqix®) and placebo (Vaseline®)

**Fig. 5:**
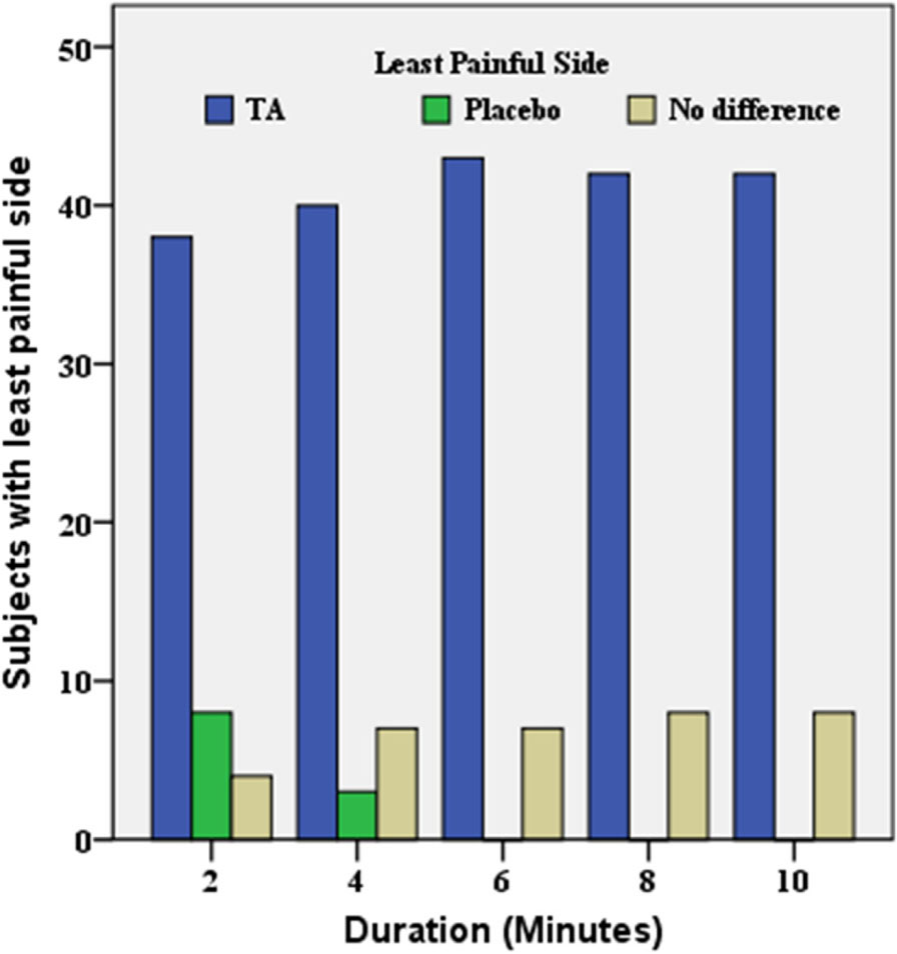
Verbal scale. A graphic representation showing the least painful side as reported by the subjects favoring either the topical anesthetic (TA Oraqix®) side or the placebo (Vaseline®) side or reporting no difference between both sides (no difference)

The percent pain sensation was assessed on the topical anesthetic (TA), Oraqix®, side and the placebo, Vaseline®, side using VAS. In comparison with Vaseline®, Oraqix® significantly reduced discomfort/pain (Fig. 4). Although the discomfort/pain scores were reduced with time in both placebo and TA sides, the TA discomfort/pain reduction was significantly better than the placebo. The significant difference was seen from the sixth minute onward. A similar pattern of discomfort/pain response was observed for almost all of the 50 subjects on the placebo side and on the TA side (Fig. 5). A gradual fall in the pain scores was noticed with time duration. Repeated measures ANOVA with a Greenhouse-Geisser correction determined that the mean pain scores improved statistically significantly with the time duration (*F*_(1.54,42.2)_ = 40.7, *p* = 0.001), with an estimated grand mean (8.37, 95% CI 6.75–9.98) (Table 1). Also, a significant difference was found in the pain level between TA and placebo (*F* = 29.2, *p* < 0.001). The estimated marginal mean pain with TA (3.96, 95% CI 1.67–6.25) was significantly less compared to placebo (12.77, 95% CI 10.48–15.06) (Table 1).

### Least painful side as reported verbally (verbal scale)—Table 2 and Fig. 5

**Table 2:** The verbal scale displaying the number (no.) and percentage (%) of subjects reporting the placebo (Vaseline) side as the least painful side, the topical anesthesia (TA), Oraqix, side as the least painful side, and no difference between both sides concerning the least pain sensation

Minutes	Placebo (Vaseline) least painful side	TA (Oraqix) least painful side	No difference
	No. (%)	No. (%)	No. (%)
2	8 (16.0)	38 (76.0)	4 (8.0)
4	3 (6.0)	40 (80.0)	7 (14.0)
6	0 (0)	43 (86.0)	7 (14.0)
8	0 (0)	42 (84.0)	8 (16.0)
10	0 (0)	42 (84.0)	8 (16.0)

The mean pain sensation scores were significantly lower on the TA side than on the placebo side, at all of the 2-min intervals (Table 2). The verbal scale results revealed that the topical anesthetic agent, Oraqix®, reduced pain significantly better than the placebo, Vaseline®, every 2 min for 10 min. This significant difference was clearly seen from the sixth minute onwards (Fig. 5). Overall, 82% of the subjects reported least pain on the TA side, while 4.4% of them mentioned less pain on the placebo side, and the remaining 13.6% felt no difference between both sides.

At 2 min, 38 subjects reported less pain on the TA side, eight subjects described the placebo side as the least painful side, and four subjects stated no difference between the sides. After 4 min, 40 subjects reported less pain on the TA side, three subjects reported less pain on the placebo side, and seven subjects reported no difference between the sides. After 6 min, 43 subjects felt decreased pain on the TA side, none described the placebo side as the least painful side, and seven subjects reported no difference between the sides. At the eighth and tenth minutes, 42 subjects reported that the TA side was the least painful side, none of the subjects reported the placebo side as the least painful side, and eight subjects stated no difference between the sides.

### Satisfaction level and opinion on TA use (questionnaire responses)—Table 3

**Table 3:** The questionnaire comprising of eight questions set for the subjects to answer after completion of the study

Question	Placebo (Vaseline) side	TA (Oraqix) side	No difference
1. Which side was more pleasant (overall satisfaction)?		47 (94%)	3 (6%)
2. Which side tasted better?	30 (6%)	15 (30%)	5 (10%)
3. Which side was more numb?	-	50 (100%)	-
4. Which side felt more numb after 30 minutes?	-	35 (70%)	15 (30%)
5. Which side do you prefer if you will have separators placed for orthodontic treatment?	-	50 (100%)	-
6. Which topical agent side would you recommend for routine use at the orthodontic clinics?	-	50 (100%)	-
7. Which topical agent side would you recommend for children and adults?	-	50 (100%)	-
8. Where there any adverse effects the next day?	-	50 (100%)	-

1. *Overall satisfaction*
  With regards to the overall satisfaction, 47 subjects reportedly felt more pleasant with the side of the topical anesthetic, and three patients reported no difference between the sides.
2. *Taste*
  Regarding the taste preference, 15 subjects reported better taste from the topical anesthesia side, 30 subjects preferred the taste from the placebo side, and five subjects reported no taste difference between both sides.
3. *Numbing effect*
  Concerning the numbing effect, all 50 subjects reported intense numbness on the side of the topical anesthetic.
4. *Presence of numbness after 30 min*
  The subjects were asked to report any sensation of numbness after 30 min from completing the study. The data showed that 35 subjects experienced slight numbness remaining on the side of the topical anesthetic and 15 subjects reported no numbness after 30 min.
5. *Personal preference*
  Regarding the personal recommendation, all 50 subjects preferred the side that contained the topical anesthetic as the material desired for them if they were to receive separators in the future.
6. *Recommendation for use in orthodontic clinics*
  For use in orthodontic clinics, all 50 subjects favored the TA side and suggested the use of that material as a routine for orthodontic separator placement.
7. *Recommendation for adults and children*
  All 50 subjects preferred the topical anesthetic side as the material of choice for apprehensive adults and children.
8. *Adverse effects*
  The subjects were asked to report any adverse reactions experienced after 1 day from the application of the materials. All 50 subjects reported no adverse reactions of any kind the following day.

## Discussion

The results of this study showed that topical anesthesia could be a valuable tool in reducing pain and discomfort associated with several orthodontic procedures. The lidocaine/prilocaine topical anesthetic, Oraqix®, could be effective in relieving pain and discomfort related to the initial placement of orthodontic elastomeric separators.

Based on clinical experience, the placement of orthodontic separators in some patients can cause immediate initial pressure, leading to discomfort and/or pain as soon as the separator is wedged between the teeth. The patients’ degree of initial and delayed responses to the uncomfortable and painful orthodontic procedures can be attributed to several factors, including age, gender, and pain threshold, which can affect the patients’ motivation for orthodontic treatment [3, 14, 27]. Therefore, some orthodontic patients do need special consideration, making it imperative to find a method that decreases the patients’ initial discomfort and pain during the separator placement visit to ensure good compliance in the orthodontic visits.

The use of topical anesthetics was found to be involved in various orthodontic procedures for relieving discomfort and pain. A study, for instance, reported the use of a benzocaine containing wax for the relief of oral mucosal irritation caused by orthodontic fixed appliances. In comparison with the unmedicated wax commonly used in orthodontic clinics, the benzocaine-medicated wax was found to be instantly effective, and its anesthetic effect kept increasing with time [28]. A preliminary study analyzed the effect of benzocaine mucoadhesive patches on orthodontic pain caused by elastomeric separators. It was revealed that the use of 20% benzocaine patches during the first 3 days after the separator placement significantly decreased the degree of pain [29]. This finding triggered the interest to investigate the effect of the lidocaine/prilocaine topical anesthetic, Oraqix®, on pain reduction from the initial placement of orthodontic separators. It would also be interesting to study the effect of Oraqix® on pain reduction after hours or days from separator placement.

In this study, after applying Oraqix®, a waiting period of 2 min was chosen before the placement of the separators. This was due to the potency of Oraqix® as previous studies showed that it is efficient from the first 2 min after application to the vestibular and palatal mucosae [7–9]. Regarding the study’s duration, a total period of 10 min was selected as it usually takes less than 5 min for placing the orthodontic elastomeric separators and about 10 min is needed to give the instructions to the patient or guardian and to answer their questions before leaving the orthodontic clinic. It was best to have the patient relieved of any disturbing sensation during the visit to ensure proper attendance to appointments, good compliance to treatment, and a pleasant in-office experience.

Concerning the VAS results of this study, regardless of the sides the materials were applied to, it was shown that the Oraqix® side reduced discomfort/pain earlier and significantly better than the Vaseline® side (Fig. 4). The significant difference in the percentage of pain reduction between both materials was evident from the fourth and sixth minutes onwards. The early onset of action shown in this study coincided with the findings from two previous studies that compared the anesthetic effect of two lidocaine/prilocaine substances with benzocaine. Both studies showed that the lidocaine/prilocaine substances (EMLA® and Oraqix®) reduced pain significantly better than benzocaine as early as the first and second minutes after application [18–20]. In this study, after 2 min from applying the materials, some subjects reported mostly pressure and discomfort after the instant separator placement, and this sensation was probably due to the fact that 2 min was not sufficient enough to anesthetize the gingival margins and periodontal ligament. This could be due to the thickness of the gingival tissues as well as the distance of penetration of the topical anesthetic to anesthetize the periodontal ligament. Moreover, due to the fact the subjects had different pain thresholds, some subjects reported low pain scores, while others reported higher pain scores during the entire duration of the study.

The overall mean discomfort/pain score on the VAS was found to be significantly lower (*p* < 0.001) with the topical anesthetic (3.9 ± 0.49 SE) than with the placebo (12.8 ± 0.90 SE) (Table 1). As observed from the VAS graphs of both the placebo and TA sides, most subjects described the sensation as pressure discomfort giving lower percentages of discomfort/pain evaluation, while a few reported a painful perception immediately after the initial placement of the elastomeric separators, and hence reported higher percentages of discomfort/pain evaluation (Fig. 4). This supported the previous findings that patients of different age, gender, ethnicity, psychosocial background, and pain tolerance and threshold could have varying responses to discomfort and pain [14].

Some studies looked at pain from orthodontic tooth separation by registering pain responses on a VAS at three time points: T1 (before insertion of the tab), T2 (immediately after insertion), and T3 (24 h after insertion) [30, 31]. In this study, the effect of the topical anesthetic Oraqix® versus the placebo Vaseline® on discomfort or pain from the very beginning of the placement of the orthodontic elastomeric separators was analyzed. It would be interesting to monitor the effect of Oraqix® 24 h after insertion. However, this is difficult to achieve this as the duration of action of Oraqix® is about 20 min in a dry field.

In this study, the verbal scale showed that most subjects reported Oraqix® as the least painful side, and the effect was evident from the second minute of application (Table 2, Fig. 5). A few subjects did report less pain on the placebo side after 2 and 4 min. Those subjects explained that the anesthetic effect was bothersome and irritating as it was too strong. When those subjects were asked in detail about their dental history, some did mention a bad dental experience in the past with anesthetic needles, and that this sensation reminded them of this disturbing perception. Moreover, throughout the duration of the study, a few subjects reported no difference between the Vaseline® and Oraqix® sides with regard to reduction of discomfort/pain perception after separator placement. Regardless of their response in the verbal scale, by the end of this study, the subjects still preferred and recommended the use of Oraqix® for adults and children in orthodontic clinics.

Regarding the overall satisfaction, a high number of subjects felt more satisfied and pleasant with the Oraqix® side as the pressure created from the separator was relieved (Table 3). Some patients reported that the placebo side felt like there was a piece of foreign object or meat stuck between their teeth creating immediate pressure that was very annoying.

The subjects’ response to the taste was inconsistent (Table 3). More subjects seemed to favor the taste of placebo Vaseline®, which was tasteless, as opposed to the taste of Oraqix®, which was bitter. A few patients were confused which side tasted better as at the end of the study, they could not remember the taste after rinsing their mouths. Also, some of the subjects mentioned that the whole oral cavity felt bitter, which meant that some of the Oraqix® material might have been mixed with saliva despite the vigilant use of saliva ejectors. Despite the bitterness of Oraqix®, most subjects did favor the taste of it. Again, most patients could not remember the difference of taste by the end of the study. In this study, the bitter taste of Oraqix® coincided with a previous study that compared the effect of two lidocaine/prilocaine substances, Oraqix® and EMLA®, on pain reduction after palatal needle sticks. Both materials had the same composition, but EMLA® was a cream and Oraqix® existed as a gel. The study showed that Oraqix® was more bitter than EMLA® as reported by the subjects [20]. Hence, since Oraqix® is FDA approved and registered for intraoral use, it was essential to suggest a recommendation to the manufacturer to improve the taste of Oraqix® to be more acceptable by patients, particularly children.

All subjects reported more numbness on the Oraqix® side, and the anesthetic effect was described as intense (Table 3). Seventy percent of the subjects mentioned that the anesthetic effect lingered with a reduced effect for 30 min after completion of the study, while 30% of the subjects reported complete absence of anesthetic effect after 30 min. As reported in one study about the onset and duration of action as assessed by probing of pocket depths, Oraqix® provided anesthesia after an application time of 30 s, with a mean duration of action of about 17 to 20 min [32]. The anesthetic effect of Oraqix® might eliminate the need for a preemptive administration of a systemic analgesic, and it may possibly limit the patient to only a postoperative dose of systemic analgesic after the anesthetic effect wears off.

When the subjects were asked about their personal preference, recommendation for routine use at orthodontic clinics, and recommendation for use for adults and children, all subjects reported the Oraqix® side as the preferred side (Table 3). This suggested that even though some subjects did report no difference between the sides in terms of overall satisfaction, they still felt that it would be of benefit for them as well as others. Moreover, all subjects reported no adverse effects experienced on both sides 1 day after the study (Table 3). As concluded in one study, in terms of the systemic effects after the application of Oraqix® in periodontal pockets, there was a large safety margin. The plasma profiles of lidocaine and prilocaine following a single dose of Oraqix® to patients with advanced periodontitis were low as compared to those reported to cause initial signs of CNS toxicity [32].

In this study, there were some weaknesses that need to be addressed in future studies. Increasing the sample size and including more males would be advantageous besides facilitating the investigation of the effect of age, gender, ethnicity, and psychosocial factors on the outcome of the subjects’ response to pain and discomfort related to placement of orthodontic elastomeric separators. It would also be motivating to explore new means to extend the effect of topical anesthetics in relieving pain and discomfort experienced after a couple of hours or even a day from the placement of orthodontic elastomeric separators.

## Conclusions

This study showed that the lidocaine/prilocaine topical anesthetic, Oraqix®, could potently relieve discomfort or pain experienced after the initial placement of the orthodontic elastomeric separators. This method could be useful for patients with a low pain threshold as well as apprehensive adults and children.
